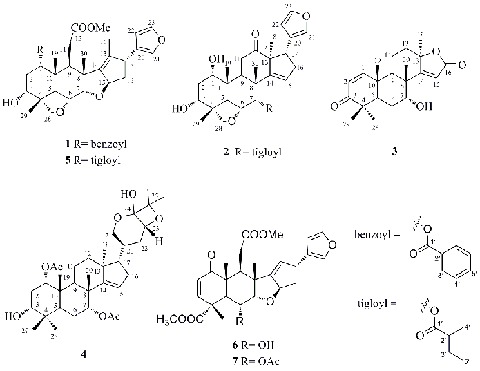# New triterpenoids from the kernels of *Azadirachta indica*

**DOI:** 10.1007/s13659-013-0005-z

**Published:** 2013-02-28

**Authors:** Hong-Wei Wang, Jie-Qing Liu, Jin-Xiong Chen, Yuan-Feng Yang, Yu-Xin Yan, Zhong-Rong Li, Ming-Hua Qiu

**Affiliations:** 15 State Key Laboratory of Phytochemistry and Plant Resources in West China, Kunming Institute of Botany, Chinese Academy of Sciences, Kunming, 650201 China; 25 University of Chinese Academy of Sciences, Beijing, 100049 China

**Keywords:** *Azadirachta indica*, triterpenoid, limonoids

## Abstract

Three new limonoids (**1**–**3**) and a new intact triterpenoid (**4**), along with three known constituents (**5**–**7**), were isolated from the dried kernels (after extracting azadirachtin) of *Azadirachta indica*. The structures of the new compounds 1-benzoyl-3-deacetyl-1-detigloyl salannin (**1**), 7-tigloyl-12-oxo vilasini (**2**), azadiralactone (**3**) and azadirahemiacetal (**4**) were elucidated by means of spectroscopic analysis. The cytotoxities of these isolated constituents were assayed.

## Introduction

Plants of the Meliaceae family are rich sources of limonoids which are structurally diverse and biologically significant.[1] *Azadirachta indica* A. Juss., as one of the most popular pesticidal plant and traditional Indian medicine, was distributed throughout Asia, Africa, and other tropical parts of the world.[2–4] During the past four decades, more than 200 compounds, including terpenoids,[1] flavones,[5] steroids,[6] coumarins[7] and polysaccharides[8] had been isolated from different parts of *A. indica*, and some of them showed the significant bioactivities such as insecticides,[1,9,10] antifungal,[1,3] antitumor[1,2,11,12] and antimalarial.[1,13,14]

Azadirachtin, as a well known natural pesticide,[3,15] was always extracted from the kernels of *A. indica*. However, we recently noticed that the kernals after extracting azadirachtin were always disposed as waste without good use. Up to now, few studies were carried out on the chemical components of the kernel residues. With the aim of searching for natural compounds with biological activities, we have investigated the kernels (after extracting azadirachtin) of *A. indica* collected from Mangshi Bright Neem Industry Developments Ltd., Yunnan Province of China. As a result, four new triterpenoids (**1**–**4**), along with three known constituents (**5**–**7**), were isolated from the dried kernels of *A. indica*.

## Results and Discussion

The MeOH extract of the kernels of *A. indica* was suspended in $H_2O$ and successively partitioned with petroleum ether and $CH_2Cl_2$. The $CH_2Cl_2$-soluble fraction was subjected to a series of chromatographic methods, and led to the isolation of seven triterpenoids (**1**–**7**).

Compound **1** was isolated as a white amorphous powder. Its molecular formula $C_{34}H_{40}O_8$ was deduced on the basis of its positive HREIMS peak at *m/z* 576.2720 $[M]^+$ (calcd for 576.2723). The IR spectrum of **1** showed absorption bands at 3446 and 1717 $cm^{-1}$ due to hydroxyl and carbonyl functionalities, respectively. The UV absorption exhibited maximum at 202 and 225 nm. The 1D NMR data (Table 1) displayed a methoxyl group [$\delta_C$ 50.7; $\delta_H$ 2.70 (3H, s)] and a benzoyl group [$\delta_C$ 165.3 (qC, C-1'), $\delta_C$ 130.1 (qC, C-2'), $\delta_C$ 129.4 (CH, C-3'), $\delta_C$ 128.5 (CH, C-4') and $\delta_C$ 133.2 (CH, C-5'); $\delta_H$ 8.06 (2H, br. d, $J$ = 8.0 Hz, H-3'), $\delta_H$ 7.50 (2H, t, $J$ = 7.5 Hz, H-4') and $\delta_H$ 7.61 (1H, t, $J$ = 7.5 Hz, H-5')]. Meanwhile, other

*To whom correspondence should be addressed. E-mail: mhchiu@mail.kib.ac.cn

Springer

**Table 1. $^{1}$H and $^{13}$C NMR spectroscopic data of compounds 1 and 2 (400 and 100 MHz; $CDCl_3$; $\delta$ in ppm)**

| | 1 | | | 2 | |
|---|---|---|---|---|---|
| pos. | $\delta_H$ (*J* in Hz) | $\delta_C$ | pos. | $\delta_H$ (*J* in Hz) | $\delta_C$ |
| 1 | 5.22 (t, 2.9) | 73.0 t | 1 | 3.58 (t, 3.1) | 72.3 d |
| 2 | 2.35 (m); 2.18 (m) | 31.0 t | 2 | 2.19 (m); 2.07 (m) | 31.1 t |
| 3 | 3.92 (br. s) | 70.2 d | 3 | 4.00 (br. s) | 71.9 t |
| 4 | | 44.3 s | 4 | | 43.9 s |
| 5 | 2.87 (d, 12.4) | 38.5 d | 5 | 2.66 (d, 12.7) | 38.4 d |
| 6 | 4.04 (dd, 3.14, 12.4) | 72.2 d | 6 | 4.22 (dd, 2.9, 12.7) | 72.5 d |
| 7 | 4.22 (d, 3.1 ) | 85.2 d | 7 | 5.72 (d, 2.9) | 73.6 d |
| 8 | | 48.8 s | 8 | | 44.3 s |
| 9 | 2.69 (br. d, 3.1) | 39.3 d | 9 | 3.43 (m) | 35.8 d |
| 10 | | 41.3 s | 10 | | 40.2 s |
| 11 | 2.34 (overlap); 2.18 (overlap) | 30.4 t | 11 | 2.40 (overlap); 2.30 (overlap) | 33.7 t |
| 12 | | 172.6 s | 12 | | 213.6 s |
| 13 | | 135.5 s | 13 | | 61.4 s |
| 14 | | 146.5 s | 14 | | 154.5 s |
| 15 | 5.41 (t, 7.2) | 87.6 d | 15 | 5.67 (t, 2.4) | 123.4 d |
| 16 | 2.31 (overlap); 2.14 (overlap) | 40.7 t | 16 | 2.42 (overlap); 2.28 (overlap) | 33.8 t |
| 17 | 3.58 (br. d, 8.6) | 49.0 d | 17 | 3.40 (overlap) | 42.5 d |
| 18 | 1.67 (s) | 12.9 q | 18 | 1.00 (s) | 18.9 q |
| 19 | 1.00 (s) | 14.8 q | 19 | 0.93 (s) | 15.0 q |
| 20 | | 127.0 s | 20 | | 125.2 s |
| 21 | 7.09 (br. s) | 138.1 d | 21 | 7.31 (br. s) | 140.8 d |
| 22 | 6.07 (br. s) | 110.3 d | 22 | 6.54 (br. s) | 112.5 d |
| 23 | 7.16 (br. s) | 142.6 d | 23 | 7.33 ( br. s) | 142.3 d |
| 28 | 4.19 (d, 7.5); 3.65 (d, 7.5) | 77.6 t | 28 | 3.82 (d, 7.20); 3.51 (d, 7.20) | 77.4 t |
| 29 | 1.19 (s) | 19.6 q | 29 | 1.10 (s) | 18.8 q |
| 30 | 1.32 (s) | 16.5 q | 30 | 1.13 (s) | 25.4 q |
| OMe | 2.70 (s) | 50.7 q | Tig-1' | | 167.3 s |
| Bz-1' | | 165.3 s | 2' | | 128.7 s |
| 2' | | 130.1 s | 3' | 6.89 (m) | 137.7 d |
| 3' | 8.06 (br. d, 8.0) | 129.4 d | 4' | 1.82 (s) | 11.9 q |
| 4' | 7.50 (t, 7.5) | 128.5 d | 5' | 1.78 (d, 7.3) | 14.2 q |
| 5' | 7.61 (t, 7.5 ) | 133.2 d | | | |

signals for 26 C-atoms in **1** were observed in the 1D NMR spectra, including seven quaternary C-atoms (three olefinic ones), eleven methines (four oxygenated ones and three olefinic groups), four methylenes, and four methyls. Detailed analysis of the $^{1}$H and $^{13}$C NMR spectral data of **1** revealed that they were similar to those of 3-deacetylsalannin (**5**).[16,17] The difference between **1** and **5** was that the tigloyl moiety in **5** was replaced by a benzoyl group in **1**.

The benzoyl group at C-1 in **1** was supported by the HMBC correlation of H-1 ($\delta_H$ 5.22) with the carbonyl group ($\delta_C$ 165.3), C-2 ($\delta_C$ 31.0), C-3 ($\delta_C$ 70.2), C-5 ($\delta_C$ 38.5), C-10 ($\delta_C$ 41.3) and Me-19 ($\delta_C$ 14.8). In the ROESY spectrum, associations of Me-19 with H-1 and Me-29 with H-3 suggested the $\alpha$-orientations of the benzoyl and hydroxyl groups. Therefore, the structure of compound **1** was elucidated as 1-benzoyl-3-deacetyl-1-detigloyl salannin.

Compound **2** was obtained as a white amorphous powder. Based on the positive HREIMS (*m/z* 524.2791, calcd for 524.2774), the molecular formula was defined as $C_{31}H_{40}O_7$. The IR spectrum showed absorption bands at 3445 (hydroxyl) and 1707 $cm^{-1}$ (an unconjugated ketone). Extensive analysis of the 1D NMR spectroscopic data (Table 1) of **2** exhibited a close resemblance with toosendone[18]. Comparison of the 1D NMR data of **2** with those of toosendone showed that the signals of the acetyl group in **2** were disappeared, and the chemical shifts of C-28 and C-6 in **2** were downfield shifted by 2~4 ppm than those in toosendone. Moreover, there was one more degree of unsaturation in **2** than in toosendone. Thus, an ether bond could be existed between C-6 and C-28. The assumption could be confirmed by the HMBC correlations between H-28 ($\delta_H$ 3.82, 3.51) with C-6 ($\delta_C$ 72.5), C-29 ($\delta_C$ 18.8) and C-4 ($\delta_C$ 43.9).

The linkage of tigloyl to C-7 was determined by the HMBC correlations from H-7 ($\delta_H$ 5.72) to C-1' ($\delta_C$ 167.3), C-6 ($\delta_C$ 72.5), C-8 ($\delta_C$ 44.3), C-5 ($\delta_C$ 38.4) and Me-30 ($\delta_C$ 25.4). Observation of the HMBC correlations of Me-18 ($\delta_H$ 1.00), H-17 ($\delta_H$ 3.40), and H-11 ($\delta_H$ 2.40 and 2.30) with carbonyl carbon ($\delta_C$ 213.6) speculated that the carbonyl carbon was located at C-12. The ROESY correlations of H-1/Me-19, H-3/Me-29 and H-7/Me-30 suggested that 1-OH, 3-OH and 7-OH were $\alpha$-oriented. Finally, the structure of **2** was deduced as 7-tigloyl-12-oxo vilasini.[19]

Compound **3** possessed a molecular formula of $C_{21}H_{28}O_4$, inferred by HREIMS at *m/z* 344.1989 $[M]^+$ (calcd for 344.1988). Its UV absorption exhibited maximum at 220 nm. The IR spectrum displayed peaks at 3347 $cm^{-1}$ (hydroxyl), 1742 $cm^{-1}$ ($\alpha,\beta$-unsaturated-$\gamma$-lactone) and 1649 $cm^{-1}$ (double-bond). The $^{1}$H NMR spectrum (Table 2) showed the presence of five methyls [$\delta_H$ 1.60 (3H, s, Me-18), 1.17 (3H, s, Me-19), 1.13 (3H, s, Me-28), 1.15 (3H, s, Me-29) and 1.31 (3H, s, Me-30)] and three olefinic protons [$\delta_H$ 7.15 (1H, d, *J* =10.3 Hz, H-1), 5.90 (1H, d, *J* =10.3 Hz, H-2) and 5.98 (1H, s, H-15)]. The $^{13}$C NMR spectra further showed the presence of one ketone ($\delta_C$ 204.7), one $\alpha,\beta$-unsaturated-$\gamma$-lactone ($\delta_C$ 172.6), one oxygenated methine ($\delta_C$ 71.2) and one quaternary carbon ($\delta_C$ 87.6). Above data suggested that **3** was the analogue of 7$\alpha$-acetoxyl-4,4,8-trimethyl-5$\alpha$-(13$\alpha$-Me)-17-oxa-androsata-1,14-dien-3,16-dione,[20] except for less of an acetoxyl functionality.

The HMBC correlations of H-5 ($\delta_H$ 2.43), H-9 ($\delta_H$ 1.76) and Me-30 ($\delta_H$ 1.31) with C-7 ($\delta_C$ 71.2) suggested that the hydroxyl group attached to C-7. The relative configuration of 7-OH was assigned to be $\alpha$-oriented by the ROESY correlation of H-7/Me-30. The ROESY correlations of Me-18 with H-9

**Table 2. $^{1}$H and $^{13}$C NMR spectroscopic data of compounds 3 and 4 (600 and 150 MHz; $CDCl_3$; $\delta$ in ppm)**

| | 3 | | | 4 | |
|---|---|---|---|---|---|
| pos. | $\delta_H$ ($J$ in Hz) | $\delta_C$ | pos. | $\delta_H$ ($J$ in Hz) | $\delta_C$ |
| 1 | 7.15 (d, 10.3) | 157.4 d | 1 | 4.88 (t, 3.0) | 74.9 d |
| 2 | 5.90 (d, 10.3) | 126.6 d | 2 | 2.28 (m); 1.95 (m) | 28.2 t |
| 3 | | 204.7 s | 3 | 3.38 (m) | 76.4 d |
| 4 | | 44.3 s | 4 | | 37.5 s |
| 5 | 2.43 (dd, 13.3, 2.6) | 44.3 d | 5 | 2.11 (m) | 36.6 d |
| 6 | 2.17 (m); 1.70 (overlap) | 27.0 t | 6 | 1.79 (m) | 23.2 t |
| 7 | 4.22 (br. s) | 71.2 d | 7 | 5.17 (t, 3.0) | 75.5 d |
| 8 | | 45.1 s | 8 | | 42.5 s |
| 9 | 1.76 (dd, 12.4, 2.2) | 46.1 d | 9 | 2.46 (dd, 12.5, 5.3) | 36.0 d |
| 10 | | 40.5 s | 10 | | 40.5 s |
| 11 | 2.03 (m); 1.66 (m) | 18.5 t | 11 | 1.42 (m); 1.26 (m) | 16.4 t |
| 12 | 2.32 (m); 1.58 (m) | 38.7 t | 12 | 1.68 (m); 1.41 (m) | 35.5 t |
| 13 | | 87.6 s | 13 | | 46.6 s |
| 14 | | 177.6 s | 14 | | 159.1 s |
| 15 | 5.98 (s) | 113.2 d | 15 | 5.29 (br. d, 2.1) | 119.8 d |
| 16 | | 172.6 s | 16 | 2.17 (m); 1.96 (m) | 33.9 t |
| 18 | 1.60 (s) | 26.8 q | 17 | 1.30 (m) | 56.9 d |
| 19 | 1.17 (s) | 19.8 q | 18 | 1.10 (s) | 20.7 q |
| 28 | 1.13 (s) | 21.8 q | 19 | 0.94 (s) | 16.2 q |
| 29 | 1.15 (s) | 27.9 q | 20 | 2.10 (m) | 31.8 d |
| 30 | 1.31 (s) | 20.8 q | 21 | 3.93 (m); 3.21 (m) | 66.2 t |
| | | | 22 | 1.83 (m); 1.28 (m) | 31.5 t |
| | | | 23 | 4.66 (br. d, 3.9) | 78.3 d |
| | | | 24 | | 95.5 s |
| | | | 25 | | 89.7 s |
| | | | 26 | 1.41 (s) | 22.9 q |
| | | | 27 | 1.28 (s) | 22.4 q |
| | | | 28 | 0.94 (s) | 28.6 q |
| | | | 29 | 0.85 (s) | 22.0 q |
| | | | 30 | 1.11 (s) | 27.2 q |
| | | | 1-O*CO*CH3 | | 169.7 s |
| | | | 1-OCO*CH3* | 2.17 (s) | 21.8 q |
| | | | 7-O*CO*CH3 | | 170.5 q |
| | | | 7-OCO*CH3* | 1.99 (s) | 21.6 q |

revealed that Me-18 was of $\alpha$-orientation. Ultimately, the structure of compound **3** was deduced and named as azadiralactone.

Compound **4** was isolated as a white amorphous solid. The molecular formula, $C_{34}H_{52}O_8$, was determined by HREIMS (*m/z* 588.3658 $[M]^+$, calcd for 588.3662). The IR spectrum revealed characteristic bands corresponding to the hydroxyl (3442 $cm^{-1}$), ester carbonyl (1736 $cm^{-1}$), and double-bond (1632 $cm^{-1}$) groups. Its UV absorption displayed maximum at 203 nm. The $^{1}$H NMR spectrum data (Table 2) showed signals for nine methyls ($\delta_H$ 0.85, 0.94, 0.94, 1.10, 1.11, 1.28, 1.41, 1.99, and 2.17). The $^{13}$C DEPT spectra exhibited 34 carbon signals, consisting of nine methyls, seven methylenes, nine methines (four oxygenated mehines and one olefinic mthine), and nine quaternary carbons (one oxygenated carbon, one olefinic carbon and one hemiacetal group). These data were consistent with the elemental formula and indicated that **4** was similar to 1$\alpha$,7$\alpha$-diacetoxyl-17$\alpha$-20*S*-21,24-epoxy-apotirucall-14-ene-3$\alpha$,23*R*,24*S*,25-tetraol.[21]

The molecular formulas of them showed the most prominent differences between them were that there was one more unsaturated unit and a $H_2O$ less in **4**. The $^{1}$H-$^{1}$H COSY correlations of H-21 ($\delta_H$ 3.93, 3.21) /H-20 ($\delta_H$ 2.10) /H-22 ($\delta_H$ 1.83, 1.28) /H-23 ($\delta_H$ 4.66), in combination with the HMBC correlations of H-21 ($\delta_H$ 3.93, 3.21) and H-23 ($\delta_H$ 4.66) with C-24 ($\delta_C$ 95.5, s), and of Me-26 ($\delta_H$ 1.41) and Me-27 ($\delta_H$ 1.28) with C-24 ($\delta_C$ 95.5, s) and C-25 ($\delta_C$ 89.7, s), respectively, deduced the tetrahydro-*2H*-pyran group between C-21 and C-24, keta group at C-24, and oxygenated carbons at C-23 and C-25. Moreover, one more unsaturated unit and a $H_2O$ less in **4** suggested an ether bond could be located between C-25 and C-23 or C-24, or between C-24 and C-23. By comparison of the chemical shifts of C-25 and C-23 with altissimanins A,[22] altissimanins B,[22] cumingianosides E[23] and cumingianosides N,[23] a conclusion can be deduced that the ether bond was between C-23 and C-25, formed an oxetane.

The HMBC correlations of H-1 ($\delta_H$ 4.88) with the ester carbonyl ($\delta_C$ 169.7), C-2 ($\delta_C$ 28.2), C-3 ($\delta_C$ 76.4), C-10 ($\delta_C$ 40.5) and Me-19 ($\delta_C$ 16.2) implied the ester carbonyl ($\delta_C$ 169.7) was attached to C-1, and of H-7 ($\delta_H$ 5.17) with the ester carbonyl ($\delta_C$ 170.5), C-5 ($\delta_C$ 36.6), C-8 ($\delta_C$ 42.5) and Me-30 ($\delta_C$ 27.2) suggested the ester carbonyl ($\delta_C$ 170.5) was assigned to C-7. Observation of ROESY correlations of H-20 with H-23 and H-17 exhibited the substituent at C-23 was of $\alpha$-orientation. 1-OAc, 3-OH and 7-OAc were determined to be $\alpha$-oriented by the ROESY correlations of Me-19/H-1, Me-29/H-3, and Me-30/H-7. Consequently, the structure of compound **4** was established and gave the name azadirahemiacetal.

Of the isolated compounds, three known compounds (**5**–**7**) were identified as 3-deacetylsalannin (**5**),[16,17] 6-deacetylnimbin (**6**),[24] and nimbin (**7**)[24] by comparing their NMR spectroscopic data with those in literature.

Compounds **1**–**7** were tested for *in vitro* inhibitory activities against HL-60, SMMC-7721, A549, MCF-7 and SW480 human tumor cell lines by the MTT method with cisplatin and taxol as positive controls.[25] However, the tested compounds didn't show the inhibitory activities with the $IC_{50}$ more than 40 $\mu$M.

## Experimental Section

**General Experimental Procedures.** Optical rotations were obtained with a Jasco P-1020 polarimeter. UV (in MeOH) and IR (in $CHCl_3$) spectra were measured on Shimadzu UV-2401 PC spectraphotometer and Bruker Tensor-27 infrared spectrophotometer, respectively. ESIMS spectra were recorded on an API QSTAR Pulsar spectrometer. EIMS and HREIMS were performed on a Waters Autospec Premier P776. 1D and 2D NMR spectra were recorded on Bruker AV-400 and Bruker Avance III-600MHz spectrometers. Chemical shifts ($\delta$) were expressed in *ppm* with reference to the TMS resonance. Semi-preparative HPLC studies were carried out on an Agilent 1100 liquid chromatograph. TLC was performed on precoated TLC plates (200–250 $\mu$m thickness, F254 Si gel 60 and F254 RP-18 Si gel 60, Qingdao Marine Chemical, Inc.) with compounds visualized by spraying the dried plates with 10% aqueous $H_2SO_4$ followed by heating until dryness. Silical gel (200–300 mesh, Qingdao Marine Chemical, Inc.), Lichroprep RP-18 (40–63 $\mu$m, Merck) and Sephadex LH-20 (20–150 $\mu$m, Pharmacia) were used for column chromatography.

**Plant Material.** Kernels (after extracting azadirachtin) of *A. indica* were provided by Mangshi Bright Neem Industry Developments Ltd., Yunnan Province of China. A voucher sample has been deposited in the State Key Laboratory of Phytochemistry and Plant Resources in West China, Kunming Institute of Botany, Chinese Academy of Sciences.

**Extraction and Isolation.** The air-dried kernels (30 kg) of *A. indica* (after extracting azadirachtin) were powdered, and extracted with MeOH under reflux. The combined extracts were concentrated under reduced pressure to give a dark brown residue. The residue was suspended in $H_2O$ (8 L), and extracted with petroleum ether (8 L × 4) and $CH_2Cl_2$ (8 L × 4). The $CH_2Cl_2$-soluble layer (1.3 kg), eluted successively with $CHCl_3$-MeOH (100:1), $CHCl_3$-MeOH (20:1), and $CHCl_3$-MeOH (5:1), was chromatographed on a silica gel column. The $CHCl_3$-MeOH (100:1) portion was evaporated to obtain a residue (215 g), which was subjected to chromatography column with petroleum ether-EtOAc (8:1, 6:1, 4:1 and 0:1) as elution, to give fractions I–VII. Fraction II was successively subjected to RP-18, Sephadex LH-20 and silica gel, and compound **6** (750.0 mg) and **7** (50.0 mg) were obtained. Fraction VI was further subjected to RP-18 chromatography column, eluting with MeOH-$H_2O$ (50:50, 65:35 and 80:20) to afford subfractions (VI-1–7). Subfraction VI-4 was subjected to HPLC using MeCN-$H_2O$ (50:50) as elution to give compound **4** (3.4 mg). Subfractions VI-2 and VI-3 were further separated and purified by silica gel chromatography column ($CHCl_3$-$Me_2CO$ 50:1, 20:1 and 5:1), to give compounds **1** (10.2 mg), **2** (7.6 mg) and **5** (700.0 mg). Fraction VII was also successively dealt with RP-18 chromatography column, silica gel chromatography column and semi-preparation HPLC, to afford compound **3** (4.2 mg).

**1-Benzoyl-3-deacetyl-1-detigloyl salannin (1):** white amorphous powder; $[\alpha]_D^{25}$ + 81.4 (*c* 0.058, MeOH); UV (MeOH) $\lambda_{max}$ (log $\varepsilon$) 225 (4.37) and 202 (4.48) nm; IR (KBr) $\nu_{max}$ 3446, 2953, 2894, 1717, 1452, 1437, 1394, 1274, 1164, 1116, 1072, 1027, and 714 cm$^{-1}$, $^1$H and $^{13}$C NMR data see Table 1; ESIMS *m/z* 599 [M + Na]$^+$; HREIMS *m/z* 576.2720 (calcd for $C_{34}H_{40}O_8$ [M ]$^+$, 576.2723).

**7-Tigloyl-12-oxo vilasini (2):** white amorphous powder; $[\alpha]_D^{25}$ – 43.0 (*c* 0.042, MeOH); UV (MeOH) $\lambda_{max}$ (log $\varepsilon$) 206 (4.28) nm; IR (KBr) $\nu_{max}$ 3445, 2960, 2931, 1707, 1648, 1457, 1441, 1392, 1263, 1156, 1138, and 1077 cm$^{-1}$, $^1$H and $^{13}$C NMR data see Table 1; EIMS *m/z* 524 [M]$^+$; HREIMS *m/z* 524.2791 (calcd for $C_{31}H_{40}O_7$ [M]$^+$, 524.2774).

**Azadiralactone (3):** white amorphous powder; $[\alpha]_D^{25}$ + 74.2 (*c* 0.047, MeOH); UV (MeOH) $\lambda_{max}$ (log $\varepsilon$) 220 (4.69) nm; IR (KBr) $\nu_{max}$ 3418, 3347, 2927, 1742, 1649, 1458, 1385, 1271, 1260, 1202, 1113, 1047 and 957 cm$^{-1}$, $^1$H and $^{13}$C NMR data see Table 2; ESIMS *m/z* 367 [M + Na]$^+$ ; HREIMS *m/z* 344.1989 (calcd for $C_{21}H_{28}O_4$ [M]$^+$, 344.1988).

**Azadirahemiacetal (4):** white amorphous powder; $[\alpha]_D^{25}$ – 60.3 (*c* 0.050, MeOH); UV (MeOH) $\lambda_{max}$ (log $\varepsilon$) 203 (3.78) nm; IR (KBr) $\nu_{max}$ 3442, 2925, 2854, 1736, 1632, 1462, 1382, 1250, and 1121 cm$^{-1}$, $^1$H and $^{13}$C NMR data see Table 2; ESIMS *m/z* 611 [M + Na]$^+$; HREIMS *m/z* 588.3658 (calcd for $C_{34}H_{52}O_8$ [M]$^+$, 588.3662).

**Cytotoxic Assay.** The anti-tumor activity of compounds **1**–**7** against HL-60, SMMC-7721, A549, MCF-7 and SW480 cell lines was determined by the MTT method.

**Acknowledgments**

The project was financially supported by the Knowledge Innovation Program of the CAS (Grant No. KSCX2-YW-G-038, KSCX2-EW-R-15), Special Foundation for National Major Basic Research (No.SB2007FY400), as well as Foundation of State Key Laboratory of Phytochemistry and Plant Resources in West China (P2010-ZZ14). The authors were sincerely grateful to Mangshi Bright Neem Industry Developments Ltd. for providing research samples, and Professor Li Yan for cytotoxicity bioassay.

**Open Access** This article is distributed under the terms of the Creative Commons Attribution License which permits any use, distribution, and reproduction in any medium, provided the original author(s) and source are credited.
